# A Synchronous Diagnosis of Metastatic Male Breast Cancer and Prostate Cancer

**DOI:** 10.1177/2324709619847230

**Published:** 2019-05-03

**Authors:** Leila Moosavi, Phyllis Kim, An Uche, Everardo Cobos

**Affiliations:** 1 Kern Medical Center, Bakersfield, CA, USA; 2 Kaiser Permanente Medical Center, Los Angeles, CA, USA; 3 Los Angeles County Harbor–UCLA Medical Center, Torrance, CA, USA

**Keywords:** synchronous, metastatic male breast cancer, pleural effusion, prostate cancer, germline mutation

## Abstract

In this article, we present a patient diagnosed synchronously with metastatic male breast cancer and prostate cancer. This is a 63-year-old male and recent immigrant from Nigeria, who sought medical attention for progressively worsening of shortness of breath and acute progression of a chronic right breast mass. An invasive breast carcinoma was diagnosed by the core biopsy of the right breast mass. Within 2 months of his breast cancer diagnosis, the patient also was diagnosed with prostate adenocarcinoma after being worked up for urinary retention. By presenting this patient with a synchronous diagnosis with metastatic male breast cancer and prostate cancer, history of chronic right breast mass, and gynecomastia, we speculate on possible cancer etiologies and risk factors.

## Introduction

Male breast carcinoma is a rare diagnosis and represents 1% of all breast cancer diagnosed each year. Although rare, male breast carcinoma incidences appear to be increasing over time.^1^ In contrast, prostate cancer is the second most common cancer in men worldwide, and the current lifetime risk of prostate cancer for men living in the United States is estimated to be approximately 1 in 6.

## Case Report

**Figure 1.**
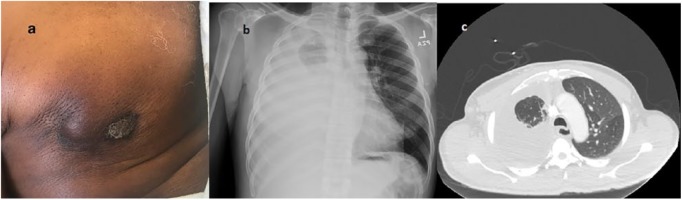
(a) Right chest breast mass, (b) chest X-ray, and (c) computed tomography of chest (prior to thoracentesis).

The patient is a 63-year-old male who recently emigrated from Nigeria. He had shortness of breath and acute progression of a chronic breast mass. The patient reported having a right chest wall/breast mass since childhood but noticed significant worsening for several months prior to being seen (Figure 1a). The mass had become enlarged, firm and tender to the touch, and was associated with overlying skin changes. The patient had also noticed a new mass in the ipsilateral axilla as well as an unintentional weight loss of 15 pounds over the past year. Further history was also notable for urinary retention and frequency for the past few months.

The physical examination revealed a remarkable large 4-cm firm, fixed right breast mass with skin thickening and retraction. The patient also had firm right axillary lymphadenopathy, diminished right-sided breath sounds throughout the entire right lung field, and prominence of the left breast.

**Figure 2.**
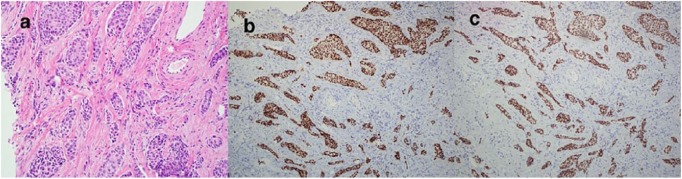
Core biopsy of the right breast mass. (a) Invasive ductal carcinoma, (b) estrogen receptor positive, and (c) GATA-3 antibody staining pattern: GATA-3 antibody appears to have an essential role in controlling genes that are involved in differentiation and proliferation of breast cancer.

An admission chest X-ray was notable for complete opacification of the right lung compatible with a large pleural effusion (Figure 1b). Further workup with computed tomography scan of the chest revealed a 6 to 7 cm mass in the right breast with right axillary adenopathy, a large right pleural effusion, and a 7-mm soft tissue nodule at the left lung base (Figure 1c). Mammography could not assess the right breast due to the inability to obtain adequate compression. However, it revealed marked gynecomastia of the left breast. The patient underwent a diagnostic and therapeutic thoracentesis, with pleural fluid studies consistent with an exudative effusion. Cytology revealed metastatic adenocarcinoma of breast primary. The patient underwent an ultrasound-guided core biopsy of the right breast mass with pathology revealing invasive ductal carcinoma, grade 2, and positive for estrogen receptor (ER) 90%, progesterone receptor (PR) 1% to 5%, and human epidermal growth factor receptor 2 positive, equivocal by immunohistochemistry, and positive by FISH (fluorescence in situ hybridization; Figure 2a-c).

For the urinary retention and frequency, a prostate-specific antigen was obtained and elevated to 122.8 ng/mL. A prostate biopsy was done and revealed prostatic adenocarcinoma, Gleason grade 8(5+3).

Due to his symptomatic pleural effusion, the patient was started on treatment for his triple-positive metastatic breast cancer with docetaxel, trastuzumab, and pertuzumab every 3 weeks with a plan for 6 cycles in total. He was also started on hormonal therapy with tamoxifen. With regard to his prostate cancer, the patient was treated with androgen deprivation therapy with leuprolide every 6 months.

## Discussion

Tumors are considered synchronous when the cancers occur at the same time or within 2 months of each other. This patient has been synchronously diagnosed with a rare cancer, metastatic male breast cancer, as well as prostate cancer. Prostate cancer is the second most common cancer diagnosis among men. Interestingly, the patient has a chronic history of a right breast mass and is found to have gynecomastia. This raises some interesting questions about the origins and pathogenesis of these 2 cancers. A review of the existing literature shows that both prostate and breast cancers are typically hormone-dependent tumors and have remarkable underlying similarities including etiology, epidemiology, and treatment approaches.

It can be postulated that this patient harbors a mutation predisposing him to malignancies. HBOC (hereditary or genetic predisposition to female breast and ovarian cancers) is well reported, linked to BRCA1 and/or BRCA2 genetic mutations.^2^ Germline mutations in the BRCA2 gene is associated with higher risk of developing breast carcinoma in comparison to men with breast cancer in the general population^3^ and prostate cancer that was diagnosed before the age of 65 years.^4^ Thus, a genetic referral and at the very least a BRCA testing is warranted. The patient is awaiting his genetics consultation at the time of this report. Arguing against a hereditary cancer syndrome is the fact that this patient lacks a strong family history of cancer.

Interestingly, the patient recently emigrated from Nigeria, where the incidence of male breast cancer is higher than in other parts of the world. The rate of breast cancer in Tanzania and areas of central Africa accounts for up to 6% of cancers in men, while male breast cancer represents between 0.5% and 1% of all breast cancers diagnosed each year in the United States and the United Kingdom.^5^ Agrawal et al explain that higher rates of male breast cancer in central and eastern Africa may be related to endemic hepatic infectious diseases that lead to high levels of estrogen.^6^ No convincing data were found that gynecomastia is associated with male breast cancer.^6^ El-Gazayerli and Abdel-Aziz elaborate similar mechanism in Egypt by explaining that increased rate of male breast cancer in this area is related to liver damage from schistosomiasis, which results in a state of hyperestrogenism.^7^

Alteration of estrogen to testosterone ratio is another possible explanation for the increased risk of hormone-sensitive cancers. Reviewing the literature demonstrates that the association between Klinefelter’s syndrome and male breast cancer is well documented. Patients with Klinefelter’s syndrome are known to have testicular dysgenesis, gynecomastia, low testosterone levels, increased gonadotropins, and they have 20 to 50 times higher risk of breast cancer in comparison to men with 46 XY.^8^ Additionally, men with mumps orchitis, undescended testes, or cirrhosis of the liver are prone to have higher risk of breast cancer due to either androgen deficiency or excess estrogens.^8^ Our patient is not known to harbor a known testicular condition or a chronic liver disease. Sasco et al in a meta-analysis study showed that the there is a significant increase of breast cancer in men who never married, or with benign breast disease, gynecomastia, Jewish ancestry, or history of breast cancer in first-degree relatives.^5^

Thellenberg et al explain that following prostate cancer therapy, the risk of endocrine-related second primary cancers such as male breast cancer and the small intestine carcinoids is increased.^9^ However, our patient is not known to have any history of previous chemotherapy prior to this presentation.

Male breast cancers are known to have higher rates of hormone receptor expression in comparison to female breast cancer. Men with breast cancer have 90% ER expression and 81% expression of PR.^8^ Also, it is reported that the chance of HER2 proto-oncogenic overexpression is less likely in male breast cancer.^8^ Almost 11% of male breast cancers are reported to have both HER2 gene amplification and protein overexpression based on the study by Rudlowski et al.^10^ Chavez-Macgregor et al describe that the distribution of tumor subtypes was different from that reported for men and also is different by race and ethnicity.^11^ Tumor-negative tumors and ER-positive/PR-negative tumors are more common in non-Hispanic black men in comparison to white men.^11^ Interestingly, our patient is a non-Hispanic black man who harbors HER2 overexpression. Male breast cancer, especially HER2-positive breast cancer, is an area that needs further investigation to determine the best treatment algorithms.

It is postulated that oncogenic viruses may play a role in human breast cancer. The 3 viruses most cited are mouse mammary tumor virus-like sequences (MMTV-LS), Epstein-Bar virus, and oncogenic (high risk) types of human papilloma virus.^12^ Though an interesting hypothesis, the reported literature does not provide support in the role of viruses as a cause of breast cancer. Johal et al report that MMTV expression may be hormonally dependent and not breast cancer-specific.^13^ The more recent literature using next-generation sequencing technologies fail to support an oncogenic viral infection as a cause of breast cancer.^14^

## Conclusion

This is a challenging and rare case of male metastatic breast cancer with synchronous prostate cancer. This patient is a recent immigrant from Nigeria and has had a chronic right breast mass since childhood with marked gynecomastia. This patient lacks a family history of breast cancer without any known testicular disease. Etiology of such a synchronous case is not well understood, while known risk factors for both of these hormone-sensitive cancers have been well identified. To the best of our knowledge, there are limited case reports with the concurrence of breast and prostate cancers reported previously.^9,15-17^ However, owing to the rarity of these type of cancers occurring synchronously, epidemiologic evidences are scant, and some of these suggested associations are controversial.^9^ As more cases of synchronous tumors are investigated, we may be able to gain a better understanding of the etiology and the underlying mechanism.
